# A Functional Variant in the Aquaporin-3 Promoter Modulates Its Expression and Correlates With Resistance to Porcine Epidemic Virus Infection in Porcine Intestinal Epithelial Cells

**DOI:** 10.3389/fmicb.2022.877644

**Published:** 2022-06-13

**Authors:** Haifei Wang, Zhenbin Bi, Kaiyu Dai, Pinghua Li, Ruihua Huang, Shenglong Wu, Wenbin Bao

**Affiliations:** ^1^Key Laboratory for Animal Genetics, Breeding, Reproduction and Molecular Design, College of Animal Science and Technology, Yangzhou University, Yangzhou, China; ^2^Institute of Swine Science, Nanjing Agricultural University, Nanjing, China; ^3^Joint International Research Laboratory of Agriculture and Agri-Product Safety, The Ministry of Education of China, Yangzhou University, Yangzhou, China

**Keywords:** porcine epidemic diarrhea virus, AQP3, promoter genetic variant, CEBPA, virus resistance

## Abstract

Porcine epidemic diarrhea virus (PEDV) causes a highly contagious intestinal disease in neonatal pigs. Aquaporin-3 (AQP3) plays important roles in maintenance of intestinal barrier function and regulation of immune responses. However, the roles of AQP3 in mediating PEDV infection to host cells and the regulatory mechanisms of AQP3 expression remain poorly understood. Here, we identified one 16 bp (GGGCGGGGTTGCGGGC) insertion mutation in the AQP3 gene promoter in Large White pigs, with the frequencies of 49.3% of heterozygotes and 31.3% of mutant homozygotes. Functional analysis by luciferase activity assay indicated that the insertion mutation results in significant enhancement in AQP3 transcriptional activity (*P* < 0.01). Mechanistic analysis showed that the inserted sequence adds binding sites for transcription factor CEBPA, which promotes the expression of AQP3. Downregulation of AQP3 by shRNA silencing in porcine intestinal epithelial cells revealed obvious increases in genome copies and viral titers of PEDV. Expression of proinflammatory cytokines (IL-6, IL-8, and IL-18) and interferons (IFN-α and IFN-β) were significantly reduced (*P* < 0.01) in AQP3 knockdown cells upon PEDV infection. Furthermore, decreased level of ZO-1 protein was also detected in AQP3 knockdown cells in response to PEDV infection. Our findings suggested a previously unknown mechanism linking the effects of promoter genetic variants on the expression of AQP3, revealed the roles of AQP3 in response to PEDV pathogenesis, and indicated the potential associations of the 16 bp insertion mutation with resistance to PEDV infection in porcine intestinal epithelial cells.

## Introduction

Porcine epidemic diarrhea virus (PEDV) is a type of alphacoronavirus that leads to porcine epidemic diarrhea with the symptoms of acute diarrhea, vomiting, and severe dehydration. PEDV infects pigs of all ages especially suckling piglets and could cause 80–100% mortality of infected suckling individuals. Porcine epidemic diarrhea has become a global disease that results in substantial economic losses for the worldwide pig industry every year (Carvajal et al., 2015). PEDV genome encodes four membrane protein and three non-structural proteins, of which the spike protein interacts with cell surface receptor for virus entry and ORF1a/b are involved in viral genome replication and transcription (Kocherhans et al., 2001). PEDV inhibits interferon signaling by influencing the expressions or activities of immune related proteins to escape the antiviral responses of host cells (Zhu et al., 2017; Wang C. et al., 2020). Genomic divergence and association analyses identified a group of single nucleotide polymorphisms (SNPs) associated with PEDV resilience (Bertolini et al., 2017), which provides candidate genetic markers for selective breeding of pigs resistant to porcine epidemic diarrhea. As the complexity of interactions between PEDV and host cells, the identification of functional regulators is highly necessary for better understanding the pathogenesis of PEDV and further developing prevention and control strategies against PEDV infection.

The aquaporin-3 (AQP3) gene is widely expressed in various epithelial cells and encodes a protein that functions as water channel and promotes the transport of non-ionic small solutes such as glycerol and hydrogen peroxide (Verkman, 2011). AQP3 plays important roles in the maintenance of intestinal epithelial barrier function through its capacity to influence the expression of tight junction proteins and to transport the bioactive molecules (Yde et al., 2021). It has been also reported that AQP3 is involved in TNF-α-induced NF-κB activation in intestinal epithelial cells and skin keratinocytes (Hara-Chikuma et al., 2015; Peplowski et al., 2017) and participates in NLRP3-inflammasome activation contributing to the setting of inflammatory response (da Silva et al., 2021). AQP3 modulates innate immune responses in the colonic epithelium through mediating hydrogen peroxide transport (Thiagarajah et al., 2017). In addition, inhibition of AQP3 function in the colon results in diarrhea (Ikarashi et al., 2012). Our previous work also showed that the expression of AQP3 was remarkably decreased in the intestinal segments of PEDV-induced piglets (Wu et al., 2020), which indicates AQP3 was a potential player in modulating PEDV-induced diarrhea. However, the functions of AQP3 in cellular responses to PEDV infection and the regulatory mechanisms underlying the expression of AQP3 remain largely unknown.

In the present study, to investigate the regulatory mechanisms of porcine AQP3 gene expression and its potential roles in mediating PEDV infection, we first identified genetic variants in the promoter region of AQP3 and analyzed the effects of identified genetic variants on AQP3 expression through affecting transcription factor binding with AQP3 promoter sequences, and second we explored the roles of AQP3 expression in mediating PEDV infections. One 16 bp insertion mutation that affects binding of transcription factor CEBPA was identified, which further regulates the expression of AQP3. In addition, down expression of the AQP3 gene resulted in increased PEDV copy numbers in host cells. Our findings uncovered the roles of the AQP3 gene in modulating PEDV infection and genetic regulations of the expression of AQP3, which may contribute to our understanding of the interactions between PEDV and host cells and aid the selective breeding for porcine epidemic diarrhea resistance.

## Materials and Methods

### Ethics Statement

The animal study proposal was approved [approval number: SYXK(Su)2016-0019] by the Institutional Animal Care and Use Committee of the Yangzhou University Animal Experiments Ethics Committee. All experimental methods were conducted in accordance with the related guidelines and regulations.

### Tissue Sample Collection and Nucleic Acid Extraction

A total of 300 Large White pigs from six families were collected from Changzhou Kang Le Farming Co., Ltd. All the animals in the same farm were raised in the same feeding procedures and conditions. Approximately 2 g of ear tissue was obtained from each animal and placed in sterile tubes. The tissues were cut into small pieces with a scalpel prior to DNA extraction. Nucleic acid was isolated from ear tissue samples using the DNA isolation kit following the manufacturer’s guidelines (TIANGEN, Beijing, China). Quality control of nucleic acid samples were performed using NanoDrop 2000 Spectrophotometer (Thermo Fisher Scientific, Wilmington, DE, United States) and agarose gel electrophoresis.

### Single Nucleotide Polymorphism Identification

According to the promoter sequence of porcine AQP3 gene (Gene ID: 100126235), PCR primers were designed using Primer Premier 5.0 software. The PCR assay was conducted in 20 μl volume containing 100 ng of DNA template, 10 μl of PCR Master Mix, 10 pmol of each forward and reverse primer, and distilled water up to 20 μl. The thermal conditions were as follows: 95°C for 5 min, 30 cycles of 95°C for 30 s, 60°C for 30 s, 72°C for 30 s, and final extension at 72°C for 10 min. PCR products were checked by electrophoresis in 1% agarose gel and sequenced by Sangon Biotech (Sangon, Shanghai, China).

### Dual-Luciferase Assays

The 245 bp of DNA sequences flanking the mutant site were synthesized and ligated into the pGL3-basic plasmid between the *Mlu*I and *Hin*dIII endonuclease sites. The constructed plasmids were then transfected into *E. coli* DH5α and cultured for constructed plasmid extraction. 293T cells were seeded into 12-well plate and cultured overnight at 37°C in the humidified incubator at an atmosphere of 5% CO_2_. The constructed plasmids with mutant sequence or with wild type sequence and control plasmids were respectively transfected into the cells using Lipofectamine 3000 (Invitrogen, Carlsbad, CA, United States). After 48 h of incubation, the luciferase activity was quantified using the dual-luciferase promoter assay system (Promega, Madison, WI, United States) following the manufacturer’s protocols. Firefly luciferase activity was normalized to Renilla luciferase activity.

### ChIP-PCR

Chromatin immunoprecipitation was conducted using the Pierce Agarose ChIP kit (ThermoFisher, Waltham, MA, United States) according to the manufacturer’s protocols. The anti-CEBPA was utilized to enrich the DNA sequences binding with CEBPA protein. The enriched DNA samples were used as template and amplified using the PCR system as mentioned above. PCR primers were designed using Primer Premier 5.0 software and provided in Supplementary Table 1.

### Overexpression of the Transcription Factor CEBPA and AQP3 Gene

The CDs region of transcription factor CEBPA (Gene ID: 397307) and AQP3 (Gene ID: 100126235) was amplified by PCR with addition of the digestion sites of restriction endonucleases. The primers and recognition sequences of restriction endonucleases are listed in Supplementary Table 1. PCR products were double digested and then ligated into the linear pcDNA3.1 plasmid using T4 DNA ligase. Correct clones were validated by agarose gel and sequencing analysis. Porcine intestinal epithelial cells were transfected with the plasmids expressing CEBPA or AQP3 using the Lipofectamine 3000 reagent (Invitrogen, Carlsbad, CA, United States) following the manufacturer’s protocols, and then selected with puromycin for 1 week. The surviving cells were expanded and prepared for subsequent experiments.

### Knockdown of the AQP3 Gene Expression

The short hairpin sequences (5’-AAGTTGAAGCCCATGGAGGTGC-3’) targeting AQP3 coding sequence were designed, synthesized, and ligated into the pGPU6-GFP-Neo plasmid. In addition, a scrambled sequence targeted none of porcine gene sequence (5’-ACGTGACACGTTCGGAGAA-3’) was set as a negative control. The plasmids containing interference and control sequences were respectively transfected into porcine intestinal epithelial cells (IPEC-J2) and continuously selected with the neomycin. The surviving cells were expanded and collected for measuring the expression level of AQP3 gene by quantitative real-time PCR (qRT-PCR).

### Porcine Epidemic Diarrhea Virus Infection Experiment

The AQP3 knockdown cells, AQP3 overexpression cells, and negative control cells were seeded in 6-well plate and infected with PEDV at MOI = 1. Following 2 h of adsorption, the infected cells were washed three times with PBS and cultured in fresh medium. After 24 h of incubation, cells were collected and treated with three times of repeated freezing and thawing for isolation of PEDV genome using TRIZOL reagent (Thermo Scientific, Waltham, MA, United States) according to the manufacturer’s guidelines. Quantities of PEDV in the cells were determined by measuring the expressions of PEDV M gene and N gene using qRT-PCR and western blotting, respectively.

### QRT-PCR Assay

Total RNA of cell samples was extracted with using the TRIZOL reagent (Thermo Scientific, Waltham, MA, United States) and reverse transcribed into cDNA using the PrimerScript RT reagent Kit according to the manufacturer’s guidelines [Takara Biotechnology (Dalian) Co., Ltd, Dalian, China]. The qRT-PCR reaction system contained 10 μL SYBR Green Mixture, 0.4 μl 50 × ROX Reference Dye II, 1 μL of each forward and reverse primer, 1 μL cDNA, and 6.6 μL deionized water. The thermal conditions were as follows: 95°C for 15 s, 40 cycles of 95°C for 5 s, 60°C for 30 s. The GAPDH and β-actin genes were used as internal reference genes. Primer sequences are provided in Supplementary Table 1. Each qRT-PCR assay was performed in triplicate, and the 2^–ΔΔCt^ method was used to calculate relative gene expression level.

### Western Blotting

Cell samples were washed twice with PBS and placed on ice with RIPA lysis buffer (250 ul per well of a 6-well plate). The whole protein was isolated using protein extraction reagent, denatured by boiling for 10 min, and further separated with SDS-PAGE. The proteins were then transferred to nitrocellulose membranes, and the membranes were blocked with PBS containing 5% non-fat dry milk and 0.2% Tween 20 at room temperature. Proteins were then incubated with the corresponding antibodies anti-ZO-1 (Novus Biologicals, Centennial, CO, United States) and anti-PEDV N (Medgene Labs, Brookings, SD, United States) at 4°C overnight and then with secondary antibody at room temperature. After wash with PBS containing 0.2% Tween 20 for three times, the bands were detected using the enhanced chemiluminescence.

### Titration of Viruses

The supernatants of the PEDV-infected cells were collected at 24 h post infection and serially 10-fold diluted to 10^–7^. The diluted culture was added to Vero cells in a 96-well plate. Infected cells were cultured for 1 week and the cytopathic effect was observed every day. Viral titers were determined by 50% tissue culture infectious dose (TCID50) analysis using the Reed-Muench method.

### Statistical Analysis

Student’s *t*-test was performed to compare the statistical differences between different groups using the R program.^1^ All results are expressed as the mean ± standard derivation (SD) of three independent experiments. The statistical significance was shown as follows: *P < 0.05; ^**^P < 0.01.

## Results

### Single Nucleotide Polymorphisms in the Porcine AQP3 Gene Promoter

**FIGURE 1:**
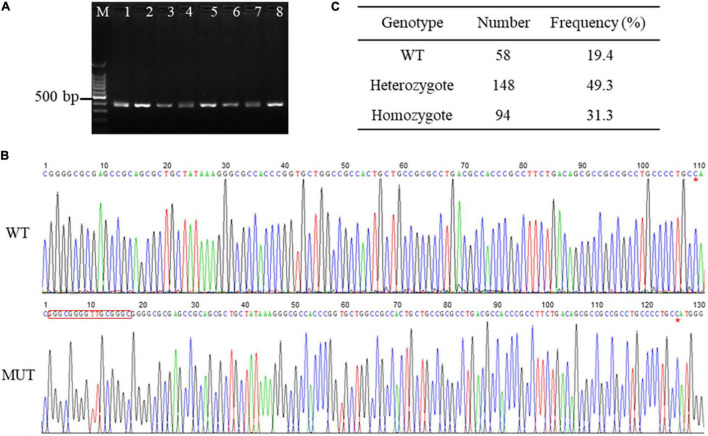
SNPs in the promoter region of AQP3 gene. **(A)** PCR products of the promoter region of AQP3 gene checked by agarose gel. M: 100 bp ladder; 1–8 represent PCR products from different samples. **(B)** SNPs identified by PCR sequencing. WT, wild type sequence; MUT, mutant sequence. The inserted sequences are marked with a red box. Red asterisk indicates the starting base of the AQP3 gene. **(C)** Genotype frequency of the insertion mutation in the Large White pigs.

To explore the genetic variations in the promoter sequence of porcine AQP3 gene, we amplified the sequence using PCR and obtained a 352 bp product (Figure 1A). Sequencing of the PCR products of AQP3 promoter sequence detected one 16 bp insertion mutation (chr10: 33045252-33045267, GGGCGGGGTTGCGGGC) located at 108 bp upstream of the AQP3 gene (chr10: 33045374-33050285) in the Large White pigs (Figure 1B), with the frequencies of 49.3% of heterozygotes and 31.3% of mutant homozygotes (Figure 1C).

### Effects of the Insertion Mutation on AQP3 Transcriptional Activity

**FIGURE 2:**
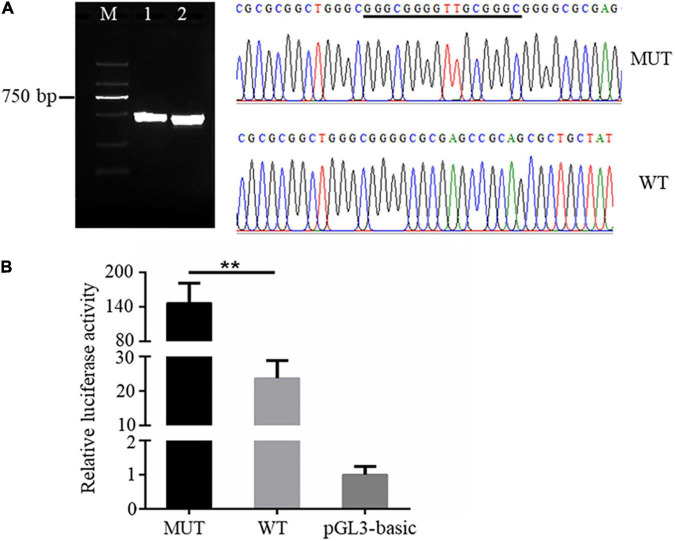
The 16 bp insertion mutation elevates promoter transcriptional activity of the AQP3 gene. **(A)** Mutant and wild type sequences verified by agarose gel (left) and sequencing (right). M: DL2000 DNA ladder; 1 and 2 separately indicates the PCR products containing the insert mutation or wild type sequences. WT, wild type sequence; MUT, mutant sequence. The inserted sequences are underlined. **(B)** Luciferase activity of AQP3 promoter constructs containing the mutant or wild type sequences. MUT, group of cells transfected with the mutant AQP3 promoter constructs; WT, group of cells transfected with the wild type AQP3 promoter constructs; pGL3-basic, group of cells transfected with the pGL3-basic empty vector. Data depicts the mean ± SD of three independent experiments; **P < 0.01.

It is known that genetic variants in the promoter region could affect the expression of downstream genes *via* altering promoter transcriptional activity (Becanovic et al., 2015). We then sought to analyze the effects of the 16 bp insertion mutation on the transcriptional activity of AQP3 promoter constructs by quantifying relative luciferase activity. The AQP3 gene promoter sequences containing the 16 bp insertion mutation and wild type sequence were amplified and verified by agarose gel and sequencing (Figure 2A). Amplicons with correct sequence was ligated with the pGL3-basic plasmid. The reconstructed plasmids containing the mutant or wild type sequence were transfected into the IPEC-J2 cells for luciferase activity analysis. Our results demonstrated that luciferase activity in cells transfected with plasmids containing the mutant sequence was significantly increased compared with the controls (Figure 2B), indicating that the 16 bp insertion mutation conferred a higher transcriptional activity.

### Insertion Mutation Promotes the Binding Capacity of Transcription Factor CEBPA to AQP3 Promoter Sequence

**FIGURE 3:**
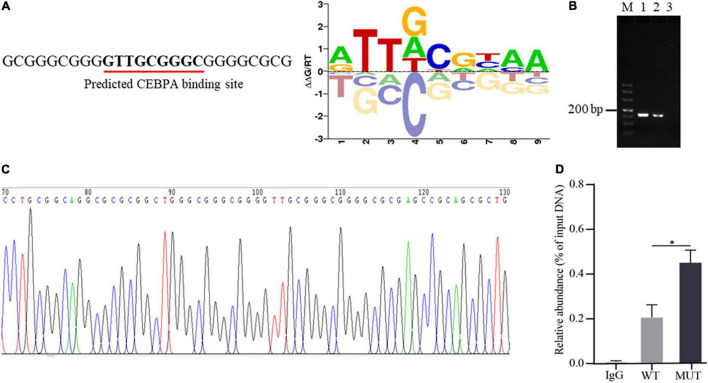
Occurrence of the insertion mutation in AQP3 promoter promotes binding capacity of CEBPA. **(A)** Prediction of transcription factor binding sites based on the sequence of the insertion mutation. The predicted binding sequence is underlined. **(B)** ChIP-PCR of the inserted sequences analyzed by agarose gel. M: DL500 DNA marker; 1: products of input DNA sample; 2: products of IP DNA sample; 3: negative control of IgG. **(C)** Sequencing of PCR products for the inserted sequences. **(D)** Enrichment of CEBPA with wild type and mutant sequences of AQP3 promoter by ChIP-qPCR. WT, wild type sequence; MUT, mutant sequence; IgG, negative control. *P < 0.05.

Altering transcription factor binding with promoter DNA sequence is one of the important ways of promoter genetic variants to mediate the expression of downstream genes (Becanovic et al., 2015; Wang Y. et al., 2020). To explore whether the 16 bp insertion mutation influences the binding of AQP3 promoter DNA sequence with transcription factors, we predicted the transcription factors potentially binding the inserted sequences. The results demonstrated that the insertion mutation has the binding sites for transcription factor CEBPA (Figure 3A). To verify the binding of transcription factor CEBPA with the inserted sequence, we performed ChIP-PCR and found that the inserted sequence was amplified (Figure 3B), and PCR products sequencing further confirmed the insertion mutation sequence (Figure 3C). Furthermore, the enrichment analysis of CEBPA binding showed that CEBPA has obviously higher binding capacity with the mutant sequences than that of wild type sequences (Figure 3D). These findings together indicated an enrichment of CEBPA occupancy at the predicted binding sites in AQP3 promoter region and the insertion mutation promotes the binding capacity of CEBPA.

### CEBPA Promotes the Expression of AQP3

**FIGURE 4:**
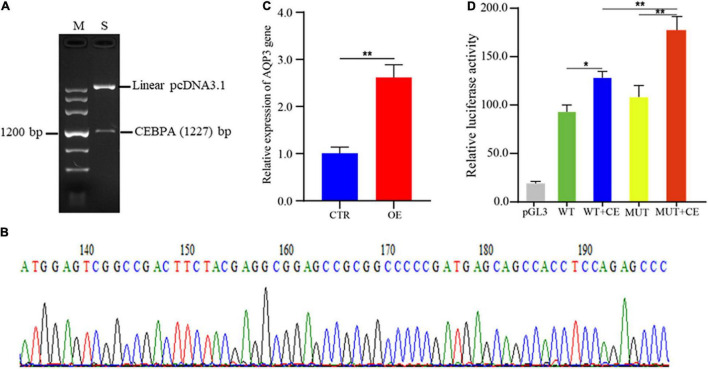
Effects of transcription factor CEBPA on AQP3 expression. **(A)** Enzyme digested products of the plasmids overexpressing CEBPA. **(B)** PCR sequencing of the plasmids overexpressing CEBPA. **(C)** Overexpression of CEBPA enhances AQP3 expression. OE, CEBPA overexpression group; CTR, control group. **(D)** Effects of CEBPA overexpression on the transcriptional activity of AQP3 promoter constructs. pGL3, cells transfected with pGL3 plasmids; WT, cells transfected with AQP3 promoter constructs containing wild type sequences; WT + CE, cells co-transfected with AQP3 promoter constructs containing wild type sequences and CEBPA overexpressing constructs; MUT, cells transfected with AQP3 promoter constructs containing the 16 bp inserted sequences; MUT + CE, cells co-transfected with AQP3 promoter constructs containing the 16 bp inserted sequences and CEBPA overexpressing constructs. The activity of each group was obtained by the ratio of firefly luciferase activity to renilla luciferase activity. Data depicts the mean ± SD of three independent experiments; *P < 0.05, **P < 0.01.

To investigate the regulatory roles of CEBPA binding on AQP3 expression, we constructed the plasmids overexpressing CEBPA. The correct plasmid clones were enzyme digested and checked by agarose gel (Figure 4A), and further validated by PCR sequencing (Figure 4B). We then assessed whether overexpression of CEBPA affects the expression of AQP3 and found that overexpression of CEBPA resulted in increased expression levels of AQP3 in porcine intestinal cells (Figure 4C). For further exploring the regulatory role of CEBPA on transcriptional activity of AQP3 gene, the AQP3 promoter constructs and CEBPA expressing plasmids were co-transfected into 293T cells. The results demonstrated that luciferase activity of MUT + CEBPA group was significantly higher (*P* < 0.01) than that of WT + CEBPA group and MUT group (Figure 4D). The luciferase activity of WT + CEBPA group was dramatically higher (*P* < 0.05) than that of WT group (Figure 4D). Our results suggested that CEBPA expression influences the transcriptional activity of AQP3 promoter and further regulates the expression of AQP3.

### AQP3 Inhibits Porcine Epidemic Diarrhea Virus Infection With IPEC-J2 Cells

**FIGURE 5:**
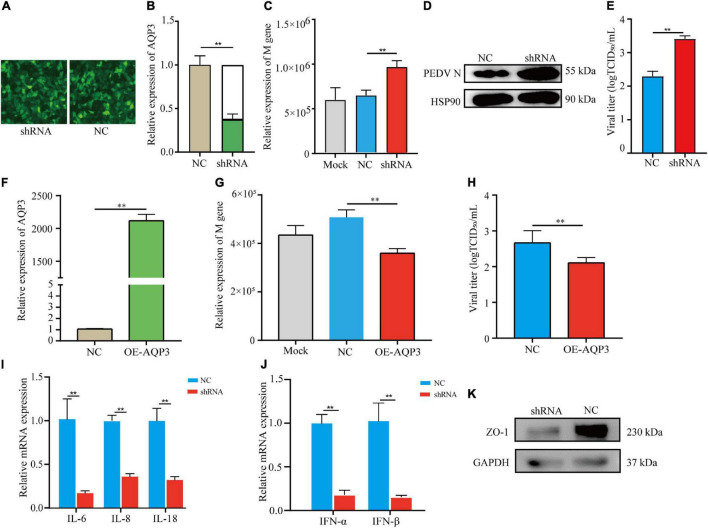
Analysis of AQP3 function in mediating PEDV infection. **(A)** Fluorescence image of cell transfected with plasmids interfering AQP3 expression. shRNA, cells transfected with plasmids containing interfering sequence targeting AQP3; NC, cells transfected with plasmids containing scramble sequence. **(B)** Relative expression of the AQP3 gene. shRNA, AQP3 knockdown cells; NC, cells transfected with scramble sequence. **(C)** Relative expression of PEDV M gene. Mock, cells without plasmid transfection; shRNA, AQP3 knockdown cells; NC, cells transfected with scramble sequence. **(D)** Western blotting analysis of PEDV N protein after 24 h of PEDV infection. shRNA, AQP3 knockdown cells; NC, cells transfected with scramble sequence. **(E)** Effects of AQP3 knockdown on viral titers determined by measuring TCID50. NC, cells transfected with scramble sequence; shRNA, AQP3 knockdown cells. **(F)** Quantification of AQP3 expression level in cells overexpressing AQP3 by qRT-PCR. NC, cells transfected with empty vector; OE-AQP3, cells transfected with vector expressing the AQP3 gene. **(G)** Relative expression of PEDV M gene. Mock, cells without plasmid transfection; NC, cells transfected with empty vector; OE-AQP3, cells overexpressing the AQP3 gene. **(H)** Effects of AQP3 overexpression on viral titers determined by measuring TCID50. NC, cells transfected with empty vector; OE-AQP3, cells overexpressing the AQP3 gene. **(I)** Expression level of proinflammatory cytokines in AQP3 knockdown and control cells post 24 h of PEDV infection. shRNA, AQP3 knockdown cells; NC, cells transfected with scramble sequence. **(J)** Expression level of type I interferons in AQP3 knockdown and control cells post 24 h of PEDV infection. shRNA, AQP3 knockdown cells; NC, cells transfected with scramble sequence. **(K)** Western blotting analysis of ZO-1 protein level in AQP3 knockdown and control cells after 24 h of PEDV infection. shRNA, AQP3 knockdown cells; NC, cells transfected with scramble sequence. Data depicts the mean ± SD of three independent experiments; **P < 0.01.

To investigate the potential functions of the AQP3 gene in mediating PEDV infection, we performed knockdown of the AQP3 gene expression by shRNA interference. The transfection efficiency of shRNA was assessed by the expression of green fluorescent protein (Figure 5A). Quantification of gene expression showed that expression of the AQP3 gene was reduced to 37.5% of the control (Figure 5B). We then infected AQP3 knockdown cells with PEDV and found that the copy numbers of PEDV in AQP3 knockdown cells was significantly increased comparing with that of controls (Figure 5C). Western blotting analysis of PEDV N protein further confirmed the increased PEDV copies in AQP3 knockdown cells (Figure 5D). In addition, AQP3 knockdown resulted in significant increases in the titer of PEDV (Figure 5E). We further overexpressed AQP3 (Figure 5F) and found that AQP3 overexpression significantly decreased the copy numbers of PEDV (Figure 5G) and the titer of PEDV (Figure 5H). These observations indicated that the AQP3 gene conferred resistance of host cells to PEDV infection. Further analysis of the effects of AQP3 knockdown on cytokine expression in response to PEDV infection displayed that proinflammatory cytokines IL-6, IL-8, and IL-18 (Figure 5I), as well as interferons IFN-α and IFN-β (Figure 5J) were significantly reduced in AQP3 knockdown cells upon PEDV infection. In addition, previous reports have demonstrated that PEDV infection results in disruptions of the barrier integrity of tight junctions (Curry et al., 2017). We then tested the changes in tight junction protein level of ZO1 in AQP3 knockdown cells upon PEDV infection and found that ZO-1 expression was obviously decreased in AQP3 knockdown cells in response to PEDV infection (Figure 5K), indicating the potentially functional interactions of AQP3 with tight junction protein ZO-1.

## Discussion

Genetic variants are important factors that influence the related phenotypes and those in the promoter regions can functionally regulate downstream gene expression through altering binding affinity of transcription factors (Becanovic et al., 2015; Mitkin et al., 2016). Genetic variants located in varied genomic regions have shown the potential as genetic markers in animal breeding programs for disease resistant and economic traits (Andersson, 2001). Herein, we identified one 16 bp insertion mutation in the promoter region of porcine AQP3 gene in Large White pigs. Further functional analysis indicated its regulatory role in AQP3 gene expression by creating binding sites for transcription factor CEBPA. We further explored the functions of AQP3 in response to PEDV infection and found the increased PEDV copies in AQP3 knockdown cells. It is known that promoter SNPs modulate pathogenic infections by controlling the expression of downstream genes involved in immune responses (Huang et al., 2007; Allen et al., 2017). Therefore, our findings indicated the potential associations of the 16 bp insertion mutation with the resistance to PEDV infection in porcine intestinal epithelial cells.

Cytokines are crucial regulators of innate immune responses to pathogenic infections. Previous studies reported that PEDV could inhibit host protein expression including proinflammatory cytokine and type I interferons through interfering the activities of NF-κb and interferon regulatory factors to suppress or evade innate immune response (Li et al., 2020). Our study demonstrated the dramatic downregulation of proinflammatory cytokines (IL-6, IL-8, and IL-18) and type I interferons (IFN-α and IFN-β) upon PEDV infection when the AQP3 gene expression was interfered. It has been revealed that AQP3 is involved in NF-κB signaling and NLRP3-inflammasome activation, and downregulated expression of AQP3 inhibits the production of cytokines such as IL-6, TNFα, and IL-1β (Hara-Chikuma et al., 2015; da Silva et al., 2021). Together, these findings support the involvement of AQP3 in host cellular immune response to PEDV infection through influencing cytokine signaling pathways. The precise mechanisms through which AQP3 affects the production of inflammatory cytokines and interferons remain further explored.

Tight junctions are important components of intestinal barrier with multiple functions in resisting the entry of xenobiotics and pathogens. Scaffolding proteins including Claudins, Occludin, and Zonula occludens are crucial molecules engaging in tight junction of epithelial cells (Zihni et al., 2016). AQP3 is involved in intestinal barrier integrity by influencing the expression of Claudin-1 and Occludin (Zhang et al., 2011). In addition, the AQP3 protein expression is mediated by protein kinase A dependent pathway (Hua et al., 2015). Activation of the protein kinase A contributes to the recruitment of ZO-1 and Claudin-1 (Köhler et al., 2004). It has been revealed that PEDV infection results in damages in tight junctions and further destroys the physiological functions of intestinal barrier (Jung et al., 2015). We herein revealed the dramatically decreased expression of the ZO-1 protein in AQP3 knockdown cells upon PEDV infection. Together, these findings suggested that AQP3 may modulate the expression of tight junction protein ZO-1 through the protein kinase A signaling pathway and further affects the intestinal barrier integrity in response to PEDV infection.

In summary, we provided functional evidence that AQP3 was involved in resistance to PEDV infection to intestinal epithelial cells and identified one insertion mutation in the promoter region, which regulates the expression of AQP3 by affecting the binding of transcription factor CEBPA. We also showed that AQP3 influences the production of proinflammatory cytokines and type I interferons upon PEDV infection. Our findings shed new light on the roles of AQP3 in response to PEDV pathogenesis and offer one 16 bp insertion mutation with potential associations with resistance to PEDV infection in porcine intestinal epithelial cells.

## Data Availability Statement

The original contributions presented in the study are included in the article/Supplementary Material, further inquiries can be directed to the corresponding authors.

## Ethics Statement

This study proposal was approved by the Institutional Animal Care and Use Committee (IACUC) of the Yangzhou University Animal Experiments Ethics Committee.

## Author Contributions

WB and SW designed the experiments. HW, ZB, and KD conducted the experiments. HW, ZB, and PL analyzed the experimental results. HW and ZB wrote the manuscript. WB, SW, and RH reviewed and modified the manuscript. All authors contributed to the article and approved the submitted version.

## Conflict of Interest

The authors declare that the research was conducted in the absence of any commercial or financial relationships that could be construed as a potential conflict of interest.

## Publisher’s Note

All claims expressed in this article are solely those of the authors and do not necessarily represent those of their affiliated organizations, or those of the publisher, the editors and the reviewers. Any product that may be evaluated in this article, or claim that may be made by its manufacturer, is not guaranteed or endorsed by the publisher.

## Footnotes

1
https://www.r-project.org/
