# Supplementary material for: A Functional Variant in the Aquaporin-3 Promoter Modulates Its Expression and Correlates With Resistance to Porcine Epidemic Virus Infection in Porcine Intestinal Epithelial Cells
Source: Front Microbiol. 2022 Jun 13;13:877644. doi: 10.3389/fmicb.2022.877644 (PMC9234456; doi:10.3389/fmicb.2022.877644)
Supplement: Supplementary file 1 [file Table_1.DOCX]

**Table S1 Primer sequences used in PCR assays**

| Symbol | Primer sequence (5’-3’) | Tm (℃) | Length (bp) | Assay |
| --- | --- | --- | --- | --- |
| AQP3  AQP3  PEDV-M  GAPDH  β-actin  CEBPA-1  CEBPA-2 | F: TCTCCTCACGCCCCATAC  R: GTCCCTCCACTCACCACG  F: TGTGACCTTCGCTATGTGCT  R: CTGTGCCGATGAACTGGTC  F: AGGTCTGCATTCCAGTGCTT  R: GGACATAGAAAGCCCAACCA  F: ACATCATCCCTGCTTCTACTGG  R: CTCGGACGCCTGCTTCAC  F: GTCGTACTCCTGCTTGCTGAT  R: CCTTCTCCTTCCAGATCATCGC  F: GGGGTACCAGACCAAGACTTGCCCTCCAC  R: CCCTCGAGTCTTCGGGTTTTGGTATCCTCA  F: CCATACCCCTACCTCCCTCAT  R: GGTGGCGCCCTTTATAGC | 58  60  60  60  60  62  56 | 352  251  216  187  119  1227  164 | PCR  qRT-PCR  qRT-PCR  qRT-PCR  qRT-PCR  PCR  ChIP-PCR |
| OE-AQP3 | F: ACGAATTCGCCACCATGGGTCGACAGAAGGAG  R: CGGGATCCCTACTCCTTGTGCTTTACTTGG | 59 | 873 | PCR |

Note: For CEBPA-1, the underlined letters represent the digestion sites of Kpn Ⅰ and Xho Ⅰ restriction endonucleases, respectively; for OE-AQP3, the underlined letters represent the digestion sites of EcoR Ⅰ and BamH Ⅰ restriction endonucleases, respectively.
